# 1,25-dihydroxyvitamin D3 ameliorates lupus nephritis through inhibiting the NF-κB and MAPK signalling pathways in MRL/lpr mice

**DOI:** 10.1186/s12882-022-02870-z

**Published:** 2022-07-08

**Authors:** Xuewei Li, Jie Liu, Yingzhe Zhao, Ning Xu, E. Lv, Chunzeng Ci, Xiangling Li

**Affiliations:** 1 Department of Rheumatology, Affiliated Hospital of Weifang Medical University, Weifang, Shandong China; 2 Affiliated Hospital of Weifang Medical University, Weifang, Shandong China; 3 Department of Nephrology, Affiliated Hospital of Weifang Medical University, Weifang, Shandong China

**Keywords:** MRL/lpr mice, 1,25-dihydroxyvitamin D3, NF-κB and MAPK signalling pathways, Lupus nephritis

## Abstract

**Background:**

Lupus nephritis (LN) is a common and serious complication of systemic lupus erythematosus (SLE). However, the aetiology and pathogenesis of LN remain unknown. 1,25-dihydroxyvitamin D3 [1,25-(OH)2-VitD3] is the active form of vitamin D, and it has been shown to perform important functions in inflammatory and immune-related diseases. In this study, we investigated the time-dependent effects of 1,25-dihydroxyvitamin D3 and explored the underlying mechanism in MRL/lpr mice, a well-studied animal model of LN.

**Methods:**

Beginning at 8 weeks of age, 24-h urine samples were collected weekly to measure the levels of protein in the urine. We treated female MRL/lpr mice with 1,25-dihydroxyvitamin D3 (4 μg/kg) or 1% DMSO by intraperitoneal injection twice weekly for 3 weeks beginning at the age of 11 weeks. The mice were separately sacrificed, and serum and kidney samples were collected at the ages of 14, 16, 18, and 20 weeks to measure creatinine (Cr) levels, blood urea nitrogen (BUN) levels, histological damage, immunological marker (A-ds DNA, C1q, C3, IgG, IgM) levels, and inflammatory factor (TNF-α, IL-17, MCP-1) levels. Furthermore, the nuclear factor kappa B (NF-κB) and the mitogen-activated protein kinase (MAPK) signalling pathways were also assessed to elucidate the underlying mechanism.

**Results:**

We found that MRL/lpr mice treated with 1,25-dihydroxyvitamin D3 displayed significantly attenuated LN. VitD3-treated mice exhibited significantly improved renal pathological damage and reduced proteinuria, BUN, SCr, A-ds DNA antibody and immune complex deposition levels (*P* < 0.05) compared with untreated MRL/lpr mice. Moreover, 1,25-dihydroxyvitamin D3 inhibited the complement cascade, inhibited the release of proinflammatory cytokines, such as TNF-α, IL-17, and MCP-1, and inhibited NF-κB and MAPK activation (*P* < 0.05).

**Conclusion:**

1,25-dihydroxyvitamin D3 exerts a protective effect against LN by inhibiting the NF-κB and MAPK signalling pathways, providing a potential treatment strategy for LN. Interestingly, the NF-κB and MAPK signalling pathways are time-dependent mediators of LN and may be associated with lupus activity.

**Supplementary Information:**

The online version contains supplementary material available at 10.1186/s12882-022-02870-z.

## Introduction

Systemic lupus erythematosus (SLE) is a complex autoimmune disease of unknown aetiology that is characterized by the production of pathogenic autoantibodies and deposition of immune complexes (ICs) [1]. Lupus nephritis (LN) is one of the most common and clinically challenging complications of SLE, and it is associated with significant morbidity and mortality. Although some effective therapies for LN have been used in the clinical setting, chronic kidney damage still occurs in many patients [2]. Even in patients who exhibit relatively better clinical responses to positive immunosuppression, kidney injury still accumulates rapidly [3]. For this reason, the development of new effective therapeutic approaches for LN remains imperative. The active form of vitamin D3, 1,25-dihydroxyvitamin D3, is reported to regulate calcium and phosphorus metabolism [4]. In recent years, 1,25-dihydroxyvitamin D3 has been shown to be an important regulator of the immune system and to have immunomodulatory properties in different inflammatory diseases [5]. It has been reported that the rate of anti-double-stranded (ds) DNA antibody positivity decreased significantly in SLE patients treated with vitamin D as a supplementary therapy [6]. Thus, in this study, we investigated the protective effects of 1,25-dihydroxyvitamin D3 and the underlying mechanism in lupus nephritis.

Animal models have played an important role in investigating the pathogenesis, progressive mechanisms, and clinical treatments of LN in vivo. The MRL/lpr mouse is universally acknowledged as a spontaneous model of LN [7], and this mouse model is characterized by massive autoantibody levels and circulating immune complexes (ICs) levels [8]. Most MRL/lpr mice develop lupus nephritis between 10 and 24 weeks of age [7].

The pathogenesis of LN is not yet clear. The presence of autoantibodies that recognize various self-molecules and overactivation of complement have been demonstrated to be related to the pathogenesis of LN [2, 3]. Anti-ds DNA antibody plays a significant role in kidney damage due to indirect or direct binding to glomerular antigens [9]. Autoreactivity to complement components may have considerable pathological consequences. The levels of C1q and C3, which are components in the complement system, are associated with the occurrence, development, and prognosis of LN [10]. In addition, IgG and IgM antibodies are key components of adaptive humoral immunity but can lead to kidney damage if they bind to self-antigens, which occurs in the autoimmune disease LN [11].

The proinflammatory cytokine tumour necrosis factor alpha (TNF-α) is produced by monocytes and macrophages and exerts a variety of physiological and pathogenic effects. It is a key factor in the initiation and perpetuation of inflammatory responses [12]. TNF-α contributes to the pathogenesis of the autoimmune disease LN [13]. In addition, interleukin-17 (IL-17) is the main signature of Th17 cells and it is also produced by double -negative (DN) T cells, macrophages, and neutrophils [14]. It has been well documented that IL-17 can induce proinflammatory gene expression both alone and in synergy with TNF-α and matrix metalloproteases [15]. IL-17 is crucial for the development of various autoimmune diseases, including LN, by promoting Th17 cell–mediated tissue inflammation [16]. In addition, chemokines are involved in the initiation and progression of LN [17]. MRL/lpr mice exhibit upregulated expression of a limited number of chemokines and chemokine receptors, such as monocyte-chemoattractant protein-1 (MCP-1), during progressive LN. MCP-1 is localized to glomeruli, tubular epithelial cells, and interstitial mononuclear cell infiltrates in the kidneys of MRL/lpr mice [18].

It has been confirmed that intracellular signalling pathways, especially the nuclear factor kappa B (NF-κB) and mitogen-activated protein kinase (MAPK) signalling pathways, are involved in the process of LN due to their ability to regulate immune responses [19, 20]. The NF-κB transcription factor is an essential regulator of numerous biological processes, including the development of the immune system, inflammation, and innate and adaptive immune responses [21]. Many studies have shown that the activation of the NF-κB pathway is related to inflammatory reactions, and autoimmune activation is also closely associated with the NF-κB pathway [22]. That is, the NF-κB pathway may play a significant role in LN. Furthermore, the MAPK signalling pathway is involved in inflammatory and autoimmune diseases. Activation of this signalling pathway can regulate many cellular functions, including cell proliferation, oxidative stress, and inflammation, leading to exacerbation of LN [20]. Given the above, the two signalling pathways play crucial roles in the processes of LN and may be important targets for LN. Thus, in the present study, we investigated the regulatory time-dependent effects of 1,25-dihydroxyvitamin D3 on LN in MRL/lpr mice and evaluated the effects on NF-kB and MAPK regulation, elucidating the mechanism underlying the effects of 1,25-dihydroxyvitamin D3 on LN.

## Materials and methods

### Animals and treatments

Forty-eight female MRL/lpr mice (weighing 23.5 ± 1.5 g at 6 weeks old) were purchased from Shanghai Laboratory Animal Center of the Chinese Academy of Sciences (Shanghai, China) and the quality certificate number: SCXK hu 2017–0005. All experiments were approved by the Institute Animal Care and Use Committee at the Weifang Medical University and carried out in compliance with the Animals in Research: Reporting In vivo Experiments (ARRIVE) guidelines. All animal procedures were in accordance with the Guide for the Care and Use of Laboratory Animals (NIH Publication No. 85–23, revised 1996). The mice were maintained under specific pathogen-free (SPF) conditions with free access to standard diet and tap water, and kept in a controlled temperature (20–24 °C) and humidity (50–55%) environment with a 12 h light-dark cycle. All the mice were acclimatized to the environment for 2 weeks prior to the experiments.

**Fig. 1:**
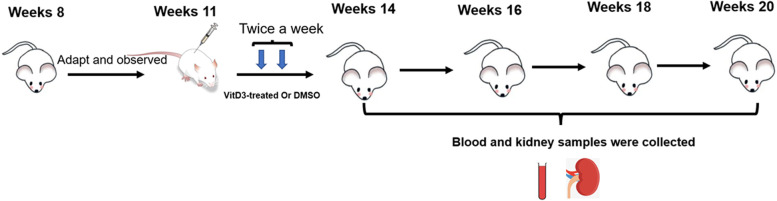
The experiment schedule

During the experiment, 24-h urine samples were collected each week starting at 8 weeks of age. At 11 weeks of age, forty-eight MRL/lpr mice were randomly divided into 8 groups, namely, the VitD3-treated groups and control groups, with 6 mice per group. The mice in the VitD3-treated groups received 4 μg/kg 1,25-dihydroxyvitamin D3 (Sigma, USA) in 1% dimethyl sulfoxide (DMSO, Sigma, USA) via intraperitoneal injection twice a week for 3 weeks; the mice in the control groups received 1% DMSO via intraperitoneal injection for 3 weeks. Then, the mice were sacrificed at 0, 2, 4, and 6 weeks after the last injection. The animals were weighed and anaesthetized with 1% pentobarbital (35 ml/kg i.p.), and blood and kidney samples were collected at planned intervals (Fig. 1). Great efforts were made to minimize the pain of animals.

### Physiological parameters

Fresh urine samples were centrifuged at 1500 rpm for 10 min at 4 °C. During the whole experimental period, the urinary protein concentrations of the supernatants from these samples were quantified weekly by an assay kit (Nanjing Jiancheng Bioengineering Institute, Jiangsu, China) and scored on a scale of 0–4: 0, negative; 1, trace; 2 (30 mg/dl); 3 (100 mg/dl); 4 (300 mg/dl); and 5 (2000 mg/dl or more). The mice were euthanized, and blood samples were collected for analysis. BUN and SCr levels were measured by using a commercial kit (Nanjing Jiancheng Bioengineering Institute, Jiangsu, China) and an Olympus AU 600 Autoanalyzer, Japan.

### Histological and immunohistochemical analyses

Kidney samples were fixed in 4% paraformaldehyde solution, embedded in paraffin, and sectioned into 5-μm slices. Haematoxylin and eosin (H&E) and Masson’s trichrome stains were used for histological analysis. After deparaffinization, antigen retrieval, and blocking with 3% H2O2 and 10% goat serum, the samples were incubated with antibodies against C1q (Beijing Solarbio Science & Technology, China) and C3 (Proteintech, China) for immunohistochemistry staining. After the secondary antibody was incubated with a biotin-streptavidin HRP conjugate, the antibody staining was visualized with DAB substrate solution. In addition, immunofluorescence was used to detect IgG (Abcam, MA) and IgM (Abcam, MA) in kidneys using fluorescent dye-conjugated antibodies against mouse IgG and the mouse IgM mu chain. All images were obtained through a microscope equipped with a colour camera (Nikon Eclipse Ni, Japan). The staining was scored by a renal pathologist blinded to the grouping of the mice. The scores of mesangial proliferation, interstitial congestion/fibrosis, C1q deposition, and C3 deposition ranged from 0 to 4 (0, absent; 1, mild; 2, mild-moderate; 3, moderate; 4, severe). The grades of crescents and tubular lesions (atrophy, casts, dilatation, and inflammatory infiltrates) were each scored from 0 to 4 (0, absent; 1, in < 25% of the section; 2, in 25–50% of the section; 3, in 50–75% of the section, and 4, in > 75% of the section). The maximum score for each mouse was 24. Five random glomeruli in each section were analysed to measure the fluorescence intensity of IgG/IgM by ImageJ.

### Enzyme-linked immunosorbent assay (ELISA) analysis

Serum samples were acquired from whole blood by centrifugation at 4000 rpm for 10 min at 4 °C and stored at − 80 °C to avoid subjecting the samples to freeze–thaw cycles until further examination. Following the manufacturer’s protocols, the serum levels of A-ds DNA, C3, TNF-α, IL-17, and MCP-1 were measured by ELISA (Wuhan Boster Bio-engineering Industrial Co., Ltd. China). The renal levels of TNF-α, IL-17 and MCP-1 were measured by ELISA (Jiangsu Meimian Industrial Co., Ltd. China).

### Western blotting analysis

Total protein was extracted from kidney samples with RIPA lysis buffer (Thermo Fischer Scientific, USA). After the BCA protein assay kit (Pierce, USA) was used to measure the protein concentrations, the protein samples were mixed with loading buffer and boiled for 5 minutes. Then, the protein samples were separated with electrophoresis gels and transferred to PVDF membranes (Millipore, USA). The membranes were incubated with the following antibodies overnight at 4 °C under agitation: NF-κBp65(1:1000, Cell Signaling Technology, USA), p-NF-κBp65(1:1000, Cell Signaling Technology, USA), IKBα(1:1000, Abcam, UK),p-IKBα(1:1000, Abcam, UK), IKKα/β (1:1000, Abcam, UK), p-IKKα/β(1:1000, Cell Signaling Technology, USA), ERK1/2 (1:1000, Cell Signaling Technology, USA), p-ERK1/2 (1:1000, Cell Signaling Technology, USA), JNK(1:1000, Abcam, UK), p-JNK(1:1000, Cell Signaling Technology, USA), p38(1:1000, Cell Signaling Technology, USA), p-p38(1:1000, Cell Signaling Technology, USA) and GAPDH (1:10,000, Abcam, UK). Then, the membranes were incubated with a horseradish peroxidase-conjugated secondary antibody for 2 h at room temperature under agitation. After the ECL Detection Kit (Thermo Fischer Scientific, USA) the used to visualized protein bands, images of the protein blots were obtained and analysed with ImageJ software.

### Real-time quantitative PCR analysis

Total RNA was isolated from kidney samples by TRIzol reagent (Takara Biotechnology, China). cDNA was generated by using a reverse transcription-polymerase chain reaction (RT–PCR) kit (Takara Biotechnology, China) following the manufacturer’s protocols. RT–PCR was carried out for A and GAPDH using a LightCycler480 II (Roche). The levels of gene expression were analysed by the following equation: 2^-(∆∆Ct)^. The PCR primer sequences are listed in Table 1.

**Table 1:** Primer sequences used in the study. (Primer Premier 5.0)

Genes	Forward primers (5′–3′)	Reverse primers (5′–3′)
NF-κBp65	CAGCATCCCTCAGCACCAT	CCATGGCTGAGGAAGGGA
p38	GACCTAAAGCCCAGCAACCT	CAGCCCACGGACCAAATAT
ERK1	GCCTTCCAATCTGCTTATCAA	GATTTGGTGTAGCCCTTGGA
JNK	CAGTTTTACAGTGTGCAGGTGG	CTTTGCGTGCGTTTGGTT
GAPDH	GGTTGTCTCCTGCGACTTCA	TGGTCCAGGGTTTCTTACTCC

### Statistical analysis

Results were expressed as the mean ± standard deviation. GraphPad Prism statistical software (Version 8, USA) was used for statistical analysis. Differences were analyzed by the t test (two groups) or two-way analysis of variance (ANOVA) (multiple groups) followed by Tukey’s post-hoc test and *p* < 0.05 was considered to be statistically significant.

## Results

### General conditions

**Fig. 2:**
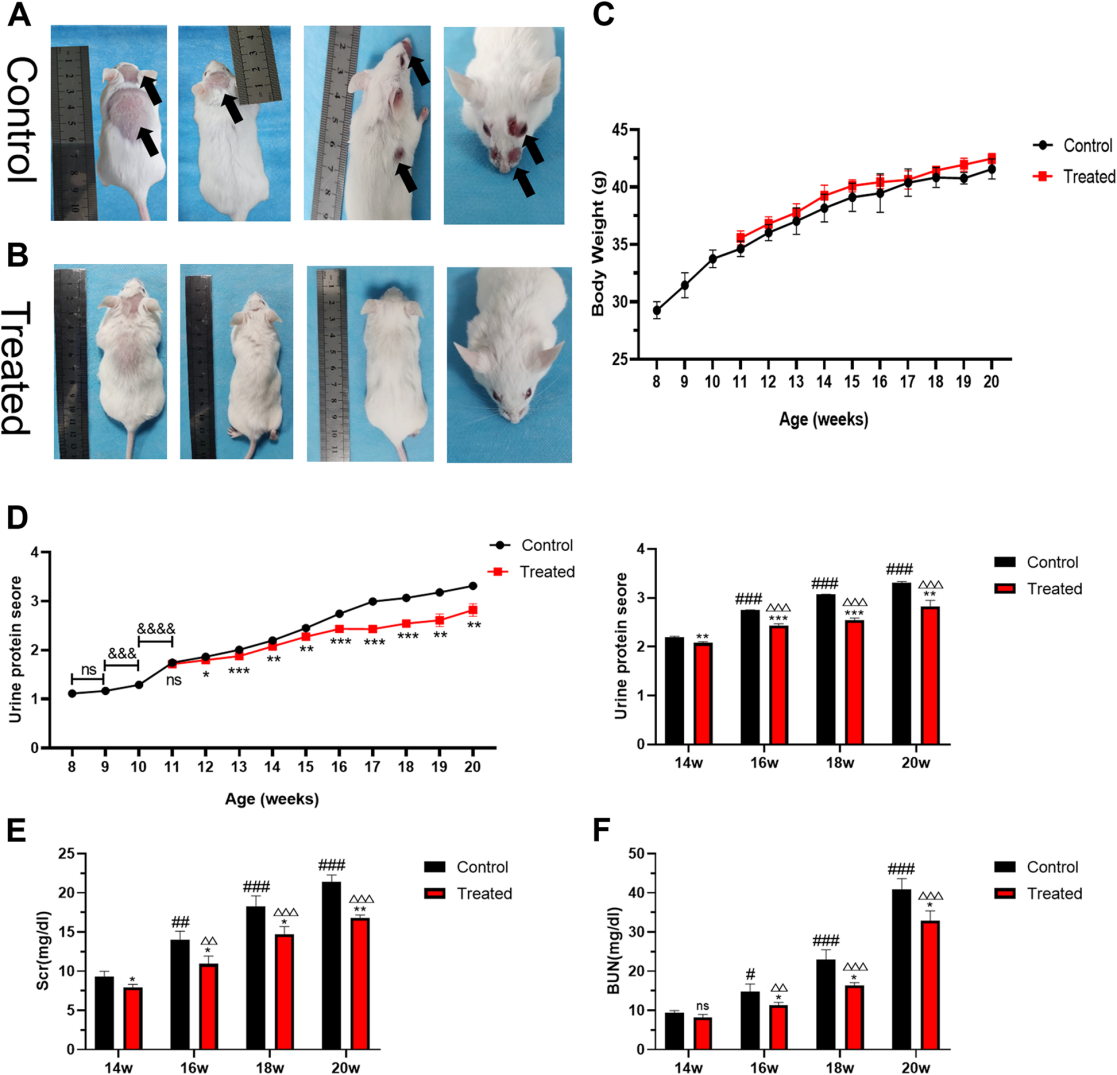
1,25-dihydroxyvitamin D3 reduced proteinuria and protected renal function. **A** MRL/lpr mice in the control groups. **B** MRL/lpr mice in the VitD3-treated groups. **C** The body weight of the experimental MRL/lpr mice. **D** Urine protein time-course studies. **E** Serum creatinine levels. **F** Blood urea nitrogen levels. Values are expressed as means ± SD (*n* = 6/group). & VS. mice at the previous week. * VS. the control group at the same time. # VS. the control group at 14 weeks. △VS. the treated group at 14 weeks. ^*#△&^ *p* < 0.05, ^**##△△&&^*p* < 0.01, ^***###△△△&&&^*p* < 0.001, ^&&&&^*P*<0.0001

Eight-week-old MRL/lpr mice began to develop different degrees of skin damage. The skin lesions of the mice in the control groups were obvious at the age of 20 weeks. In contrast, the mice treated with 1,25-dihydroxyvitamin D3 presented less skin substantial damage (Fig. 2). There were no significant differences in body weight among the groups of mice (Fig. 2), although the body weights of the VitD3-treated groups were heavier than those of the control group. One mouse in the control group died unexpectedly, and there was no unexpected death in the VitD3-treated groups before 20 weeks of age.

### 1,25-dihydroxyvitamin D3 reduced proteinuria and protected renal function

The levels of proteinuria in MRL/lpr mice were significantly elevated after 10 weeks of age, suggesting that the mice model with LN had been successfully established. During the whole period of intraperitoneal injection, the levels of proteinuria in the VitD3-treated groups were lower than those in the VitD3-treated groups at the ages of 13 weeks and 14 weeks. After 3 weeks of treatment, the levels of proteinuria in all groups of VitD3-treated MRL/lpr mice were significantly lower than those in all the groups of DMSO-treated mice (*P*s < 0.05). The protein levels in the urine of the MRL/lpr mice in the VitD3-treated groups were still not significantly elevated by 20 weeks of age. Similarly, treatment with 1,25-dihydroxyvitamin D3 significantly reduced the serum creatinine and urea nitrogen levels in the MRL/lpr mice, whereas these levels increased with time. All the data demonstrated that 1,25-dihydroxyvitamin D3 improved renal function in MRL/lpr mice (Fig. 2).

### 1,25-dihydroxyvitamin D3 ameliorated glomerular, tubular, and interstitial injuries

**Fig. 3:**
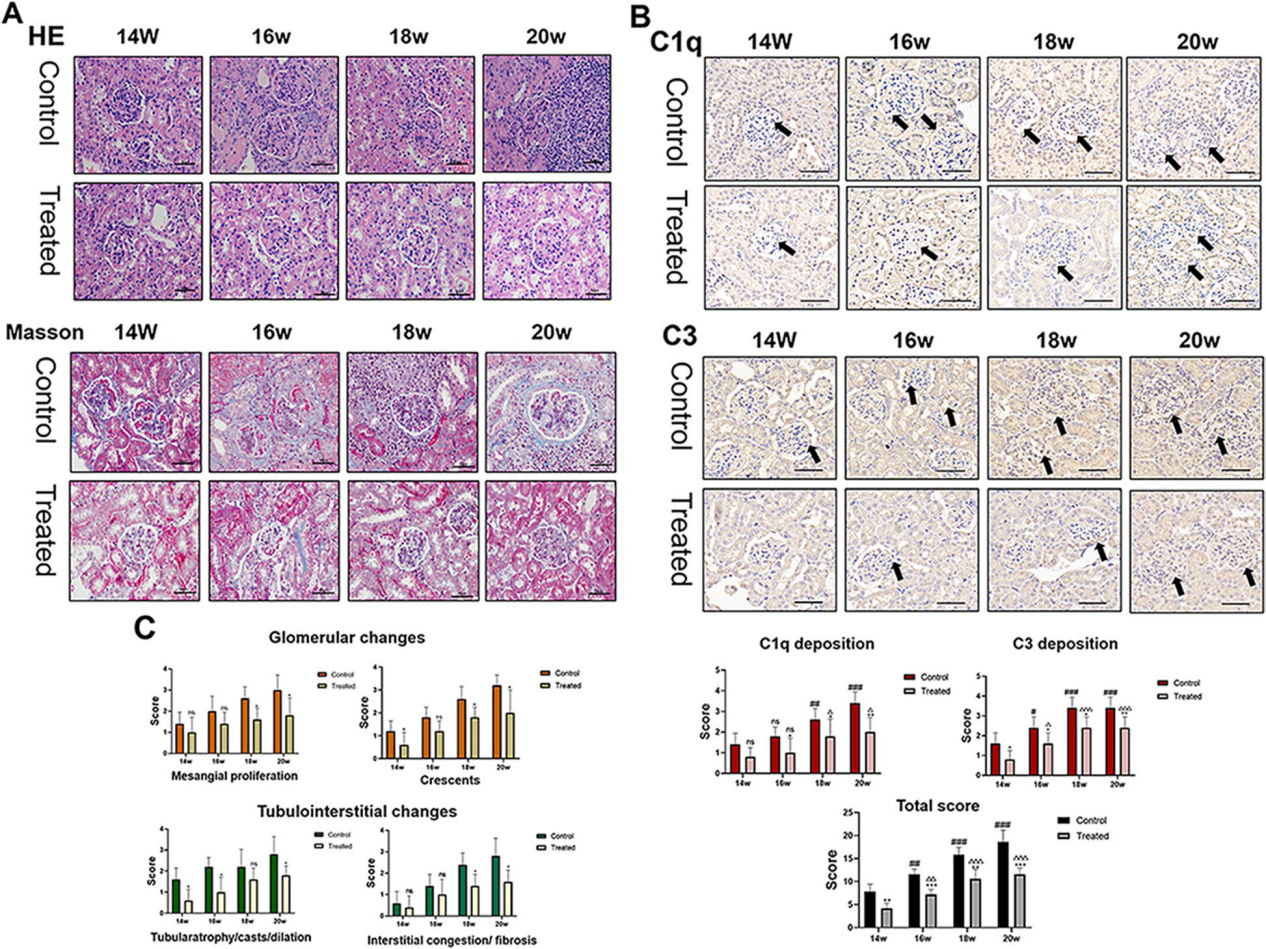
1,25-dihydroxyvitamin D3 ameliorated glomerular, tubular, and interstitial injuries. **A** Histopathological features of the renal tissues determined by HE, Masson (400×, Scale bar = 50 μm). **B** Histopathological features of the renal tissues determined by immunohistochemistry (400×, Scale bar = 50 μm). **C** Scoring results. Values are expressed as means ± SD (*n* = 3/group). * VS. the control group at the same time. # VS. the control group at 14 weeks. △VS. the treated group at 14 weeks. ^*#△^ *p* < 0.05, ^**##△△^*p* < 0.01, ^***###△△△^*p* < 0.001

Lupus nephritis was observed by HE staining and Masson’s trichrome staining. Light microscopy analyses of kidney tissues revealed pathological alterations. In Fig. 3, the staining of mouse kidneys showed that the MRL/lpr mice in the control groups had significantly more nephritis than those in the VitD3-treated groups. The main pathological features include proliferation of endothelial and mesangial cells, basement membrane thickening, infiltration of inflammatory cells, ICs deposition, and even crescent formation. According to HE staining, the control mice showed significant glomerulonephritis, an increased number of matrix and cells in the glomerulus, degeneration of renal tubular epithelial cells, necrosis of renal tubules, infiltration of numerous inflammatory cells, expansion of a small number of renal tubules, increased numbers of eosinophils and tubular type formation, renal interstitial congestion, and fibrosis. According to Masson’s trichrome staining, substantial philoazaleine protein levels were deposited in strips under the capillary endothelium, resulting in basement membrane thickening. In addition, to explore the effect of 1,25-dihydroxyvitamin D3 on the deposition of complement C1q and C3, immunohistochemistry was performed using renal tissue sections. As shown in Fig. 3 (black arrow), the depositions of complement C1q and C3 in all the VitD3-treated groups were significantly reduced compared with that in the control groups. In addition, the kidney damage of the older mice was more serious than that of the younger mice. These evidences illustrated that 1,25-dihydroxyvitamin D3 inhibits inflammatory reactions in the kidney, thus ameliorating glomerular, tubular, and interstitial injuries in MRL/lpr mice.

### 1,25-dihydroxyvitamin D3 decreased deposition of IgG/IgM

**Fig. 4:**
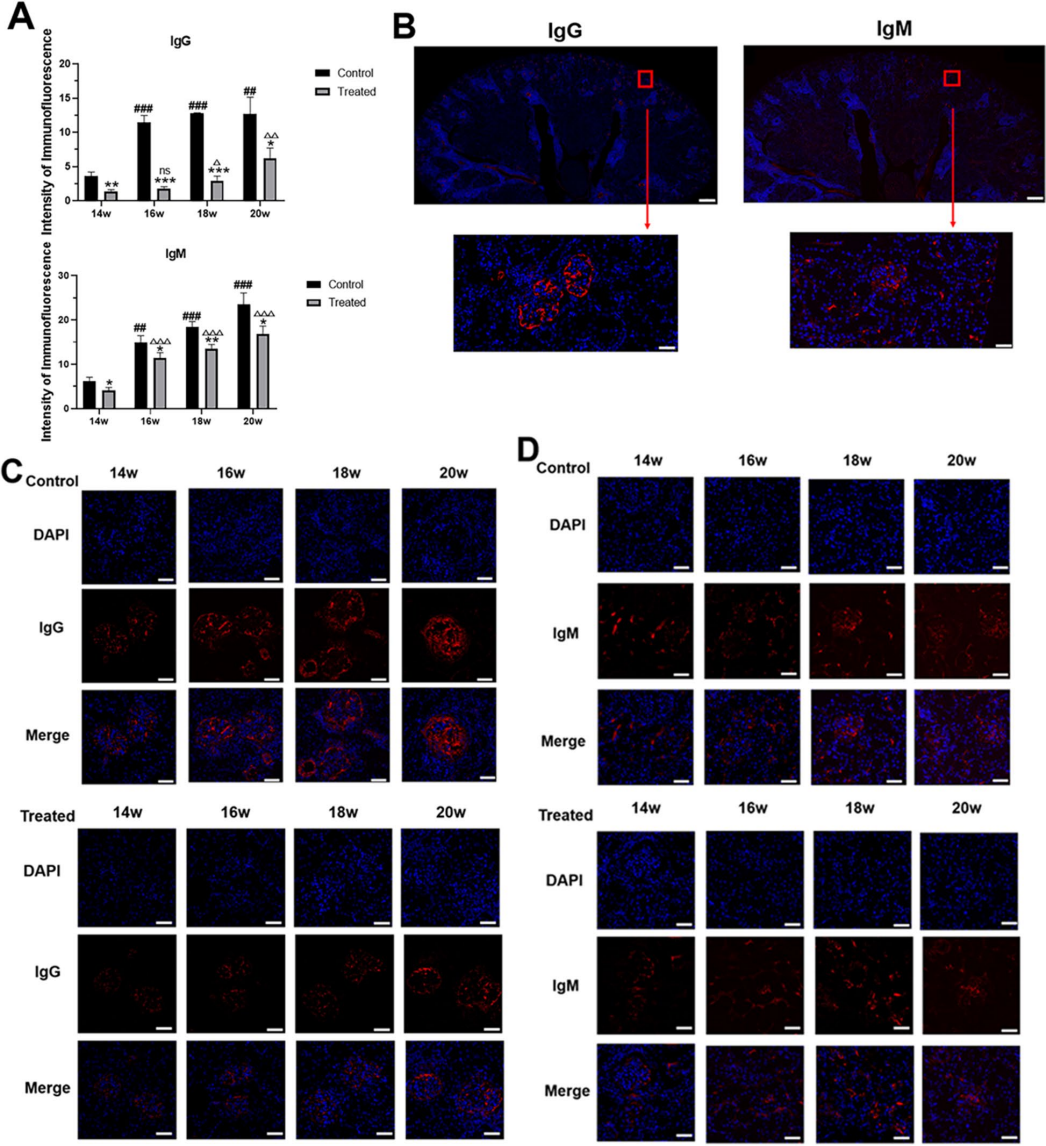
1,25-dihydroxyvitamin D3 decreased deposition of IgG/M in MRL/lpr mice. **A** The intensity of immunofluorescence. **B-D** Immunofluorescence findings for renal tissues. 3×, Scale bar = 500 μm (upper panel) and 400×, 50 μm (lower panel). Values are expressed as means ± SD (n = 3/group). * VS. the control group at the same time. # VS. the control group at T1. △VS. the treated group at T1. ^*#△^ *p* < 0.05, ^**##△△^*p* < 0.01, ^***###△△△^*p* < 0.001

The deposition of antibodies plays a crucial role in the pathogenesis of lupus nephritis. We analysed the deposition of IgG and IgM in the glomeruli of mice by immunofluorescence. We observed significantly decreased deposition of IgG and IgM in the glomeruli of the VitD3-treated mice by immunofluorescence. Furthermore, IgG/IgM depositions on the glomerulus increased with time in all the groups. These results indicated that lupus activity increased with time and that 1,25-dihydroxyvitamin D3 could alleviate lupus activity, which in turn reduced the symptoms of lupus. (Fig. 4).

### 1,25-dihydroxyvitamin D3 reduced lupus activity and inhibited proinflammatory cytokine production

**Fig. 5:**
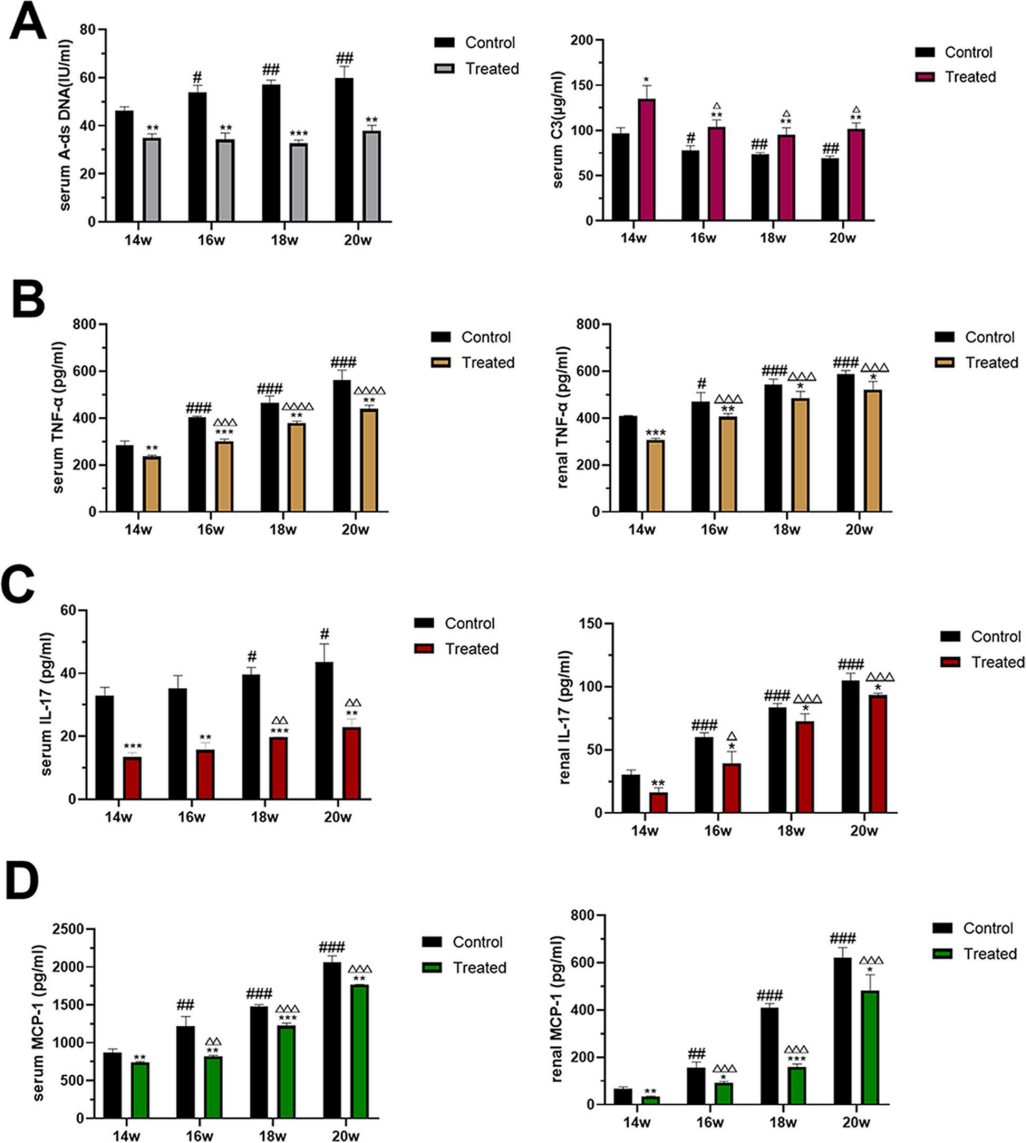
1,25-dihydroxyvitamin D3 reduced lupus activity and inhibited the pro-inflammatory cytokines. **A** The concentrations of A-ds DNA and C3 in the serum. **B-D** The concentrations of TNF-α, IL-17, and MCP-1 in the serum and kidneys. Values are expressed as means ± SD (n = 6/group). * VS. the control group at the same time. # VS. the control group at 14 weeks. △VS. the treated group at 14 weeks. ^*#△^ *p* < 0.05, ^**##△△^*p* < 0.01, ^***###△△△^*p* < 0.001

In general, the abnormal activation of autoimmunity was significantly lower in the MRL/lpr mice treated with 1,25-dihydroxyvitamin D3. From 14 weeks to 20 weeks, the serum A-ds DNA levels of the MRL/lpr mice in the VitD3-treated groups were significantly lower than those in the MRL/lpr mice in the control groups (*P* < 0.05), and there was no significant difference between the levels of the four VitD3-treated groups (Fig. 5). However, the level of serum A-ds DNA in the control groups increased with time. As shown in Fig. 5, the levels of serum C3 complement were increased in the MRL/lpr mice in the VitD3-treated groups compared to those in the mice in the control groups, whereas these levels decreased with time (*P* < 0.05).

To determine the effect of 1,25-dihydroxyvitamin D3 on such proinflammatory cytokine production, we measured the levels of TNF-α, IL-17, and MCP-1 present in the serum and kidneys. As shown in Fig. 5, the levels of TNF-α, IL-17, and MCP-1 were significantly reduced in the VitD3-treated groups compared to the control groups, whereas these levels increased with time (*P* < 0.05). The results demonstrated that lupus activity increased with time and that 1,25-dihydroxyvitamin D3 has the capacity to inhibit the excessive release of inflammatory cytokines, reducing lupus activity.

### 1,25-dihydroxyvitamin D3 inhibited the NF-κB signalling pathway

**Fig. 6:**
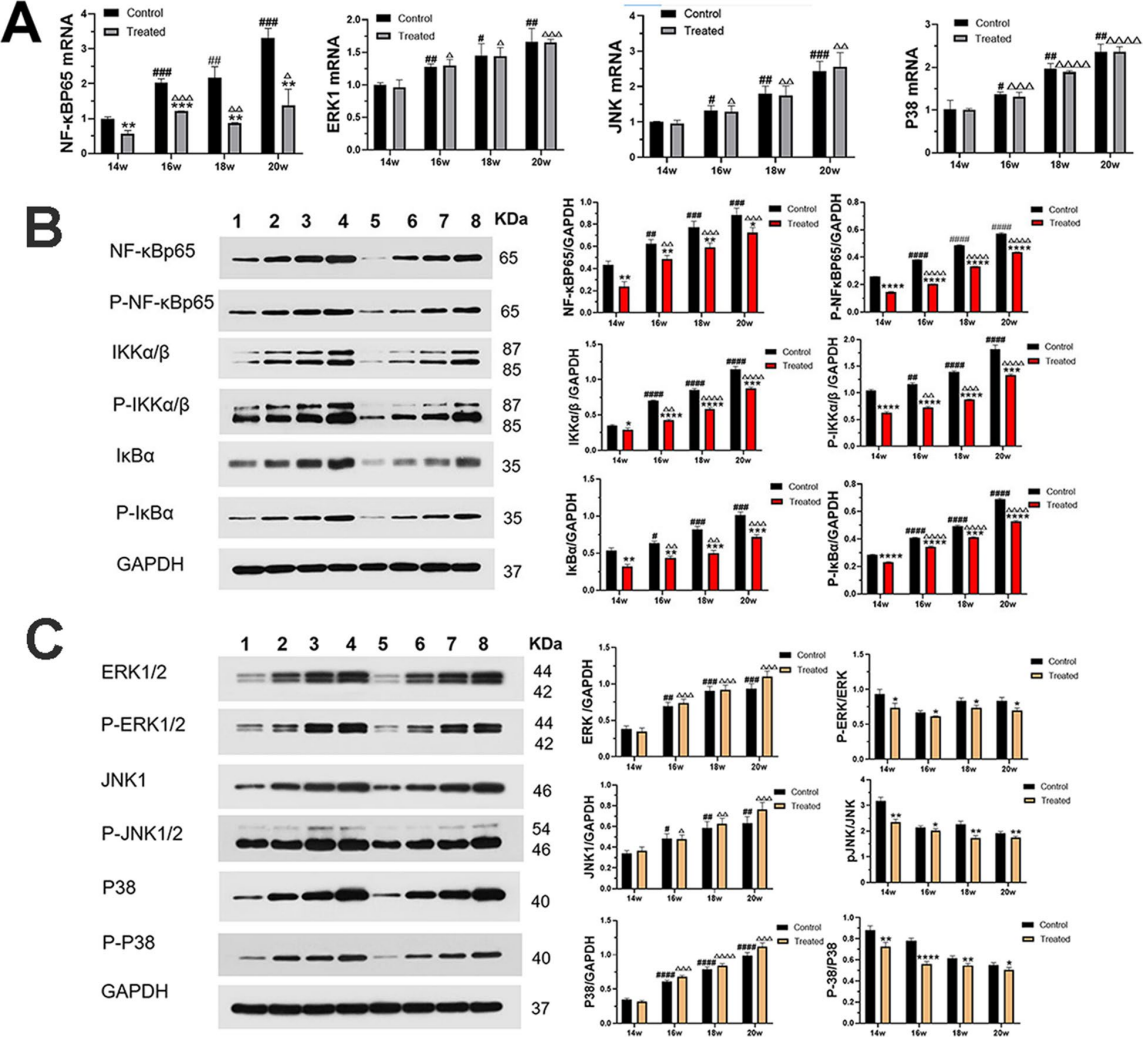
1,25-dihydroxyvitamin D3 inhibited the NF-κB/MAPKs signaling pathways. **A** The mRNA levels by RT-PCR. **B**, **C** The protein levels by Western blot, bands 1–8 represent the control groups at 14, 16, 18, 20 weeks and the VitD3-treated groups at 14, 16, 18, 20 weeks respectively. The samples derive from the same experiment and the gels/blots were processed in parallel. Data are presented as means ± SD (n = 3/group). * VS. the control group at the same time. # VS. the control group at 14 weeks. △VS. the treated group at 14 weeks. ^*#△^ *p* < 0.05, ^**##△△^*p* < 0.01, ^***###△△△^*p* < 0.001, ^****####△△△△^*p* < 0.0001

The NF-κB signalling pathway has been shown to be involved in the regulation of inflammatory and immune responses, thereby playing an important role in LN. To determine the effect of 1,25-dihydroxyvitamin D3 on the NF-κB signalling pathway, the expression of NF-κB pathway-related genes and proteins was measured over time. RT-PCR assays showed that the mRNA expression levels of NF-κBP65 in the control groups were higher than those in the VitD3-treated groups (*P* < 0.05). Similarly, as shown in Fig. 6, Western blotting analysis showed that the protein expression levels of NF-κBp65, IKKα/β, and IκB-α and the phosphorylation of NF-κBp65, IKKα/β, and IκB-α in the control groups were higher than those of the VitD3-treated groups (*P* < 0.05). In addition, the results provided interesting data on the time-dependent expression of NF-κB signalling pathway-related molecules in MRL/lpr mice. Together, these data confirmed that 1,25-dihydroxyvitamin D3 could inhibit the activation of the NF-κB pathway.

### 1,25-dihydroxyvitamin D3 blocked the MAPK signalling pathway

To further explain the mechanisms by which 1,25-dihydroxyvitamin D3 inhibits LN, we also analysed the MAPK signalling pathway in kidney tissue. Real-time PCR and Western blotting assays showed that there was no significant difference in the expression levels of ERK, JNK, and P38 between the groups at the same time points, whereas the levels increased with time (Fig. 6). However, compared to that in the control groups, the phosphorylation of ERK, JNK and P38 in the VitD3-treated groups was significantly decreased (*P* < 0.05). Additionally, the expression of MAPK signalling pathway-related molecules was significantly increased over time (Fig. 6). These results suggested that 1,25-dihydroxyvitamin D3 suppressed the phosphorylation of proteins in the MAPK pathway during LN in a time-dependent manner.

## Discussion

The aims of this study were to investigate the time-dependent effects of 1,25-dihydroxyvitamin D3 on LN in MRL/lpr mice and to evaluate the effects on the regulation of NF-kB and MAPK signalling. Our study showed that 1,25-dihydroxyvitamin D3 treatment ameliorates lupus nephritis in MRL/lpr mice, as shown by improved renal function, decreased immune complex deposition, reduced inflammatory cytokine production, and diminished NF-κB and MAPK pathway activation.

Skin lesions are one of the typical symptoms of SLE, and 72–85% of patients develop skin lesions during the course of SLE [23]. Skin lesions are characterized by epidermal atrophy, hyperkeratosis, liquefactive degeneration at the epidermal basal cell layer, infiltration of inflammatory cells and free melanin pigment [24]. It was observed that the MRL/lpr mice began to show different degrees of skin lesions at the age of 8 weeks, especially in the skin of the head, back, mouth and nose. However, 1,25-dihydroxyvitamin D3 treatment significantly attenuated the degree of skin lesion formation. The pathogenesis of skin lesions in SLE, which needs to be further investigated, may involve deposition of IgG molecules related to lupus, immune cells, cytokines, and intracellular molecules in the skin, as well as exposure to ultraviolet light [25].

Multiple factors, including genetic susceptibility and environmental effects, contribute to the pathogenesis of LN, and the production of autoantibodies, the circulation of ICs, and severe proteinuria are the most common manifestations. The effect of 1,25-dihydroxyvitamin D3 on LN was confirmed by renal pathology and immunofluorescence in MRL/lpr mice. H&E and Masson’s staining showed that 1,25-dihydroxyvitamin D3 treatment significantly attenuated the histological damage to the kidney, and the kidney scores of the 1,25-dihydroxyvitamin D3-treated mice were lower than those of the DMSO-treated mice due to reduced mesangial proliferation, basement membrane thickening, interstitial fibrosis, and inflammatory infiltration. Moreover, clinical renal remission also relies mainly on improvements in proteinuria and autoantibodies [26]. One study indicated that serum A-ds DNA concentration was closely correlated with the severity of kidney damage because glomerular collagen is bound by DNA and A-ds DNA antibodies can be eluted from nephritic kidneys [27]. Lupus is closely related to defects in apoptosis clearance [28], and studies have shown that altering the levels of autoantigen clearance could promote the production of anti-nuclear autoantibodies in SLE [29]. The dysfunction in the efficient removal of apoptotic material may be caused by failure of the autophagic process in immune cells [28]. Previous studies have revealed that VitD3 may improve macrophage phagocytic defects and increase their number [30]. This finding strongly supports our observation that 1,25-dihydroxyvitamin D3 reduces A-ds DNA antibody levels in LN. However, the effective therapies that are currently in use do not always effectively decrease the A-ds DNA antibody levels in serum. In our study, serum A-ds DNA levels were markedly increased over time after disease onset, which may be associated with lupus activity, while 1,25-dihydroxyvitamin D3 treatment notably reduced A-ds DNA antibody levels, suggesting that 1,25-dihydroxyvitamin D3 ameliorated LN and inhibited lupus activity.

A previous study suggested that not only the classical pathway but also the alternative pathway is involved in the pathogenesis of LN [31]. Complement factors are bound in the kidney with immune complex deposition or formation [32]. The deposition of C3, which is a component of the classical pathway, the alternate pathway, and the complement mannose pathway, is characteristic of LN. C1q is the first subcomponent of the C1 complex associated with the classical pathway of complement activation [33]. Due to activation by C1q and other unknown mechanisms, complement factor C3 is deposited in the glomerulus [34, 35], inducing the proliferation of glomerular mesangial cells and ultimately leading to nephropathy [36]. Circulating C3 levels are usually decreased due to its consumption, and these levels are associated with disease [35]. A recent study showed that a C3 blocker effectively improved proteinuria and renal function in MRL/lpr mice [34], which strongly suggests the importance of exploring the effects of 1,25-dihydroxyvitamin D3 on C3 levels in LN. In our study, the serum level of C3 decreased, and the deposition of C1q and C3 increased over time, which may be associated with lupus activity. Through complement profiling, our results showed that 1,25-dihydroxyvitamin D3 prevents the consumption of C1q and C3, suggesting that 1,25-dihydroxyvitamin D3 acts via the inhibition of the complement cascade to some extent. Furthermore, deposition of immune reactants also plays a pivotal role in the occurrence, development, and prognosis of LN [37]. IgG and IgM are immunoglobulins with different isotypes that recognize renal antigens. They play key regulatory roles in many biological processes, such as activation of complement cascades, regulation of gene expression and proliferation of cells [38]. They induce phenotypic changes in glomerular resident cells, leading to kidney damage in LN [39]. Additional evidence showed that 1,25-dihydroxyvitamin D3 treatment inhibited local IgG/M deposition in the kidney to protect glomerular mesangial cells. Together, these results indicated that 1,25-dihydroxyvitamin D3 was responsible for improving clinical presentations and reversing the pathological damage caused by LN.

TNF-α and its receptors are involved in the pathogenesis of autoimmune diseases, and TNF-α has both the effects of controlling autoimmunity and promoting inflammation [40]. TNF-α contributes to the pathogenesis of LN as it promotes the activation and differentiation of macrophages, and its levels are increased in active LN and correlate with disease activity [41]. IL-17 has the potential to induce the production of additional inflammatory cytokines and chemokines and to promote the recruitment of inflammatory cells, such as monocytes and neutrophils, to the inflamed organ; additionally, it is involved in the activation of many proinflammatory pathways [42]. IL-17 promotes immune hyperactivation and participates in kidney damage in LN by inducing inflammation that triggers LN development [14]. High serum levels of IL-17 have been observed in active LN and correlate with lupus activity [43]. In MRL/lpr mice, mice lacking IL-17 showed greatly improved survival and were largely protected from the development of glomerulonephritis [44]. C-C motif chemokine ligand 2 (CCL2), also known as MCP-1, and its chemokine receptor CCR2 promote LN by driving intrarenal inflammation via the recruitment and activation of proinflammatory leukocyte subsets. CCl2-deficient MRL/lpr mice are protected from LN [45]. In addition, several studies have shown that TNF-α can induce the expression of MCP-1 [46, 47] and that IL-17 can upregulate MCP-1 [48]. In our study, the levels of TNF-α, IL-17, and MCP-1were gradually upregulated with time, demonstrating that these proteins may be responsible for lupus activity. However, 1,25-dihydroxyvitamin D3 significantly reduced the levels of TNF-α, IL-17, and MCP-1 both in the serum and kidneys of MRL/lpr mice. These findings demonstrated that 1,25-dihydroxyvitamin D3 has a protective effect against LN by suppressing proinflammatory cytokine release to some extent.

To further investigate the mechanism by which 1,25-dihydroxyvitamin D3 protects against inflammation, the activation of the NF-κB pathway was examined. The NF-κB pathway is known to trigger inflammatory responses, and it controls the expression of several genes, such as proinflammatory cytokines, including TNF-α, and is essential for self-reactivity [19]. Inflammation caused by activation of the NF-κB pathway plays an important role in the pathogenesis of LN [49]. In resting cells, IκBα prevents the translocation of p65 into the nucleus. The IκB kinase (IKK) complex is activated by various stimuli and then phosphorylates and degrades the IκBα protein, releasing the p65 heterodimer to regulate the transcription of various genes [50]. Many methods of inhibiting NF-κB have demonstrated that this inhibition results in LN remission. In our study, the expression of molecules related to the NF-κB signalling pathway was markedly upregulated in the kidneys of MRL/lpr mice over time, demonstrating that activation of the NF-κB signalling pathway is an important time-dependent mediator of kidney damage in MRL/lpr and may be responsible for lupus activity. However, 1,25-dihydroxyvitamin D3 evidently inhibited the NF-κB signalling pathway. Additionally, we found that the expression of the proinflammatory cytokine TNF-α was significantly reduced in the VitD3-treated groups compared to the control groups. To a certain extent, our data further proved that the NF-κB signalling pathway and downstream proinflammatory cytokine TNF-α, which may be associated with lupus activity, were inhibited by 1,25-dihydroxyvitamin D3.

MAPK are a family of protein-serine/threonine kinases that mediate the basic biological process of inflammatory and innate immune responses, including the ERK1/2, JNK [1–3], and p38 (α, β, γ and δ) [20]. In response to inflammatory signals, MAPK are activated by the phosphorylation of ERK1/2, JNK, and p38 [51]. Furthermore, NF-κB may be downstream of MAPK, and studies have shown that the P38MAPK signalling pathway is involved in the activation of NF-κB [52]. There may be crosstalk between the signalling pathways. Under normal conditions, NF-kB is located in the cytoplasm. Once stimulated by exogenous inducers or inflammatory cytokines, NF-κB is released from the cytoplasmic complex and translocates to the nucleus to regulate the expression of inflammatory mediators [53]. Moreover, in our study, we found that the MAPK signalling pathway was also markedly activated in the kidneys of MRL/lpr mice over time, suggesting that activation of the MAPK signalling pathway is another important time-dependent mediator of LN in MRL/lpr mice and may also be associated with lupus activity. Therefore, decreasing the activation of the MAPK and NF-kB signalling pathways may be beneficial to LN. In this study, 1,25-dihydroxyvitamin D3 significantly inhibited the phosphorylation of ERK1/2, JNK1/2, and p38. Thus, our data showed that 1,25-dihydroxyvitamin D3 significantly inhibited the NF-κB and MAPK signalling pathways to ameliorate lupus activity, providing potential targets for LN treatment.

## Conclusion

In summary, 1,25-dihydroxyvitamin D3 exerted a protective effect against LN by inhibiting the NF-κB and MAPK signalling pathways, providing a potential treatment strategy and therapeutic target for LN. Moreover, the NF-κB and MAPK signalling pathways are time-dependent mediators of LN and may be associated with lupus activity.

Our study has several limitations to be resolved in the future. No antagonist group or in vitro experiment was used in this study; thus, plasmid construction will be performed in the next assay. Further research should explore in detail the effect of the VitD3 response on immune cells in LN, especially dendritic cells. In addition, we would also like to investigate more molecular mechanisms mediated by NF-kB/MAPK in our future research.

## Supplementary Information

**Additional file 1.**

## Data Availability

The datasets generated and/or analysed during the current study are not publicly available but are available from the corresponding author on reasonable request.

## Abbreviations

LN: Lupus nephritis
SLE: Systemic lupus erythematosus
1,25-(OH)_2_-VitD_3_: 1,25-dihydroxyvitamin D3
ICs: Immune complexes
DMSO: Dimethyl sulfoxide
ds DNA: Double-stranded DNA
TNF-α: Tumor necrosis factor alpha
IL-17: Interlukine-17
MCP-1: Monocyte-chemoattractant protein-1
CCL2: C-C motif chemokine ligand 2
DN T cell: Double negative T cell
NF-κB: Nuclear Factor kappa B;
MAPK: Mitogen-activated protein kinase
Scr: Serum creatinine
BUN: Blood urea nitrogen
