# Supplementary material for: 1,25-dihydroxyvitamin D3 ameliorates lupus nephritis through inhibiting the NF-κB and MAPK signalling pathways in MRL/lpr mice
Source: BMC Nephrol. 2022 Jul 8;23:243. doi: 10.1186/s12882-022-02870-z (PMC9264719; doi:10.1186/s12882-022-02870-z)

Fig 6B

1. Scanned files of original, unprocessed images

NF-κB 65KDa

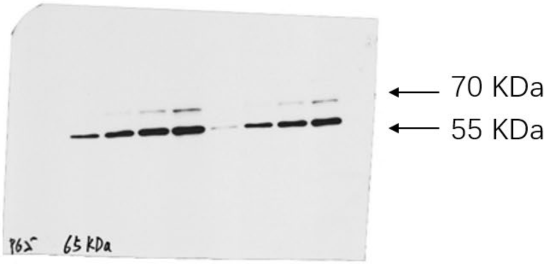

P-NF-κB 65KDa

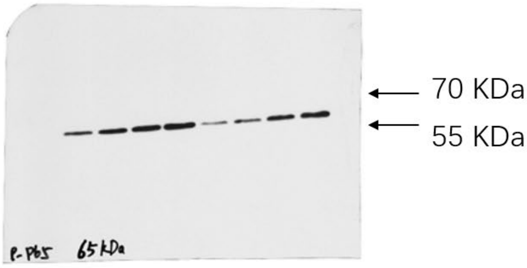

IKBα 35KDa

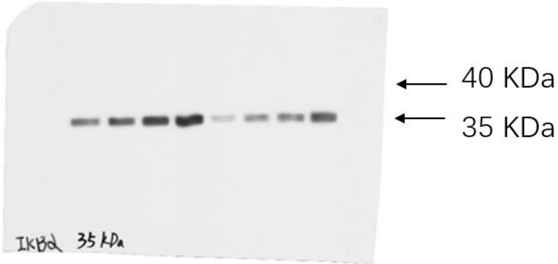

P-IKBα 35KDa

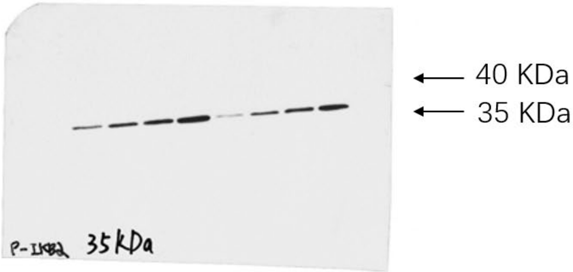

IKKα+IKKβ 85/87KDa

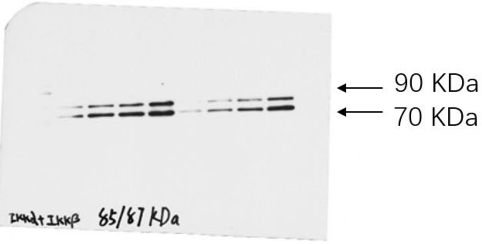

P-IKKα+IKKβ 85/87KDa

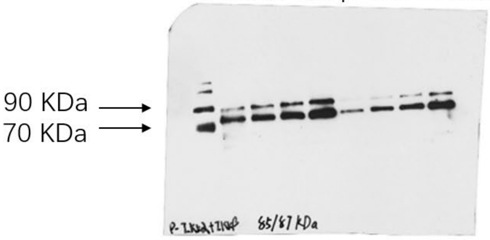

GAPDH 37KDa

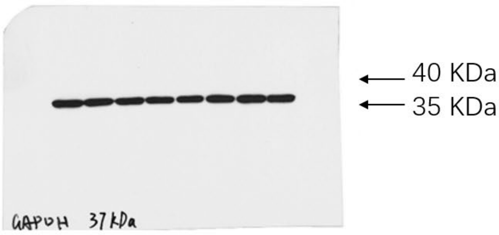

2. X-ray films of the original, unprocessed images

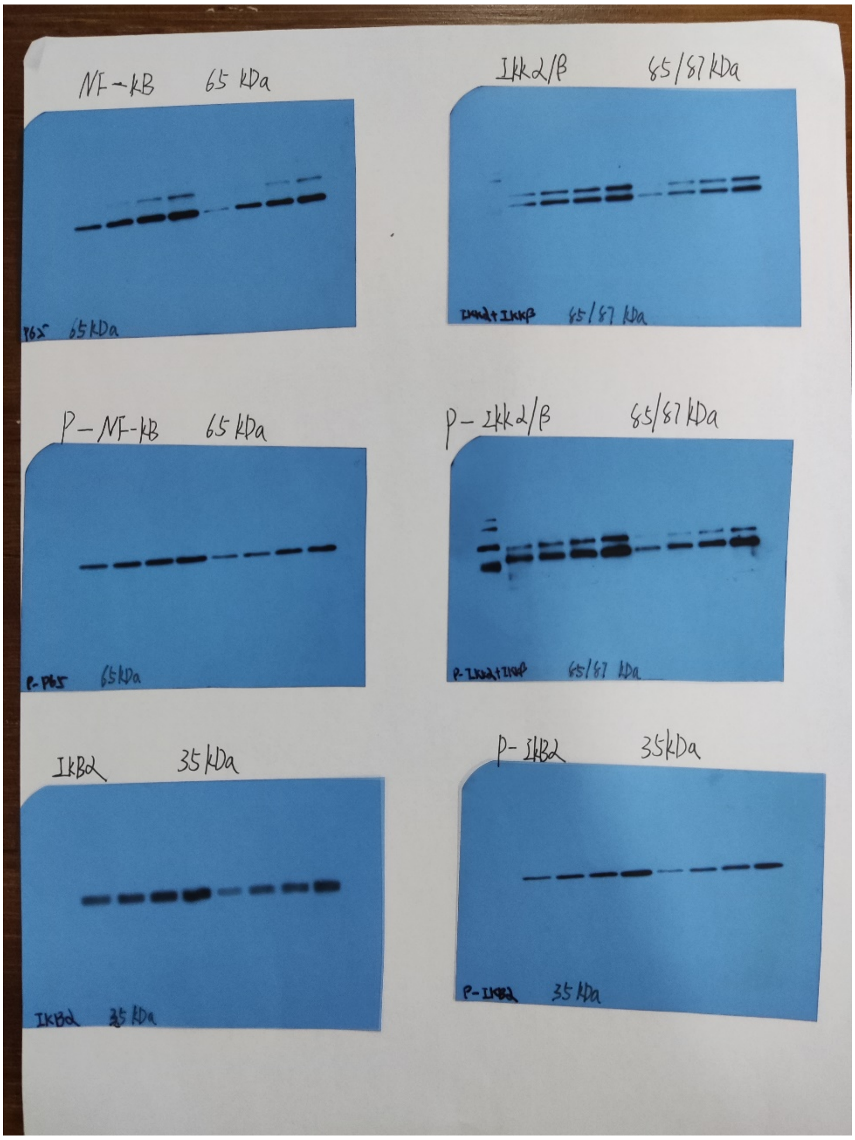

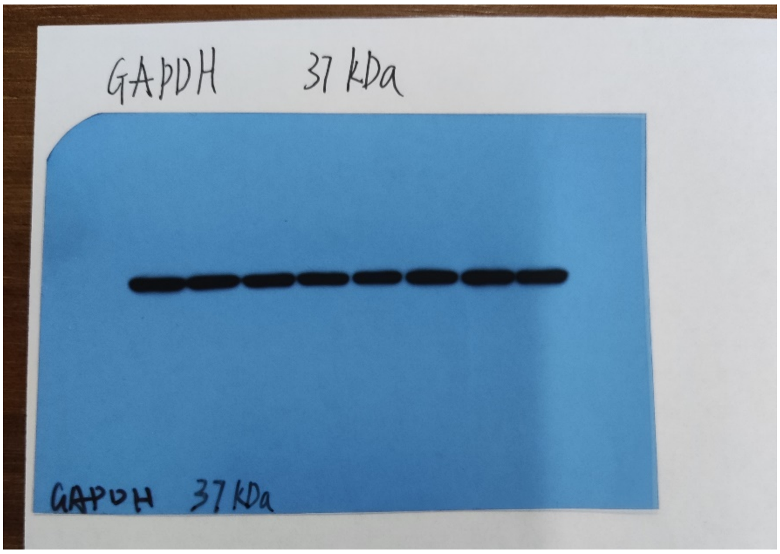

Fig 6C

1. Scanned files of original, unprocessed images

P38 40KDa

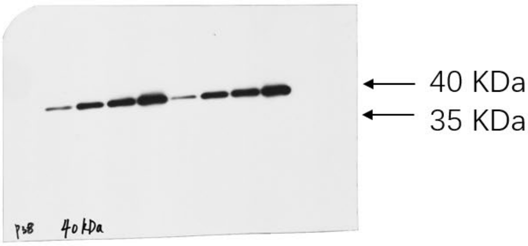

P-P38 40KDa

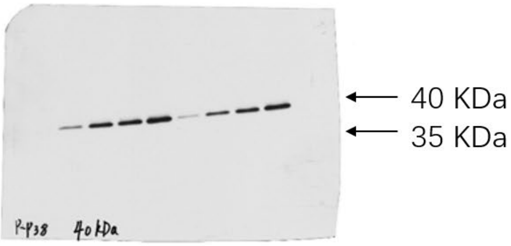

ERK1/2 42/44KDa

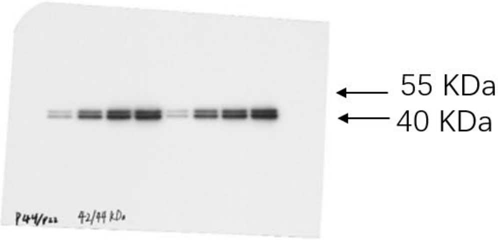

P-ERK1/2 42/44KDa

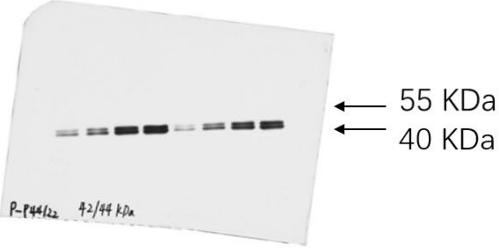

JNK1 46KDa

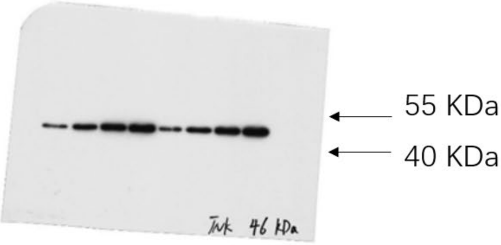

P-JNK1/2 46/54KDa

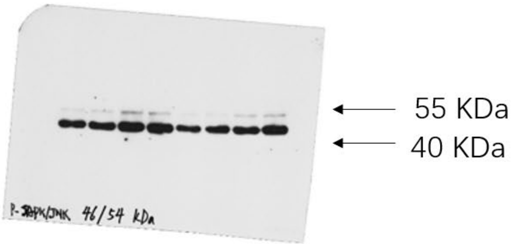

GAPDH 37KDa

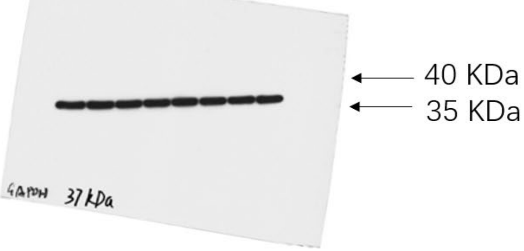

2. X-ray films of the original, unprocessed images

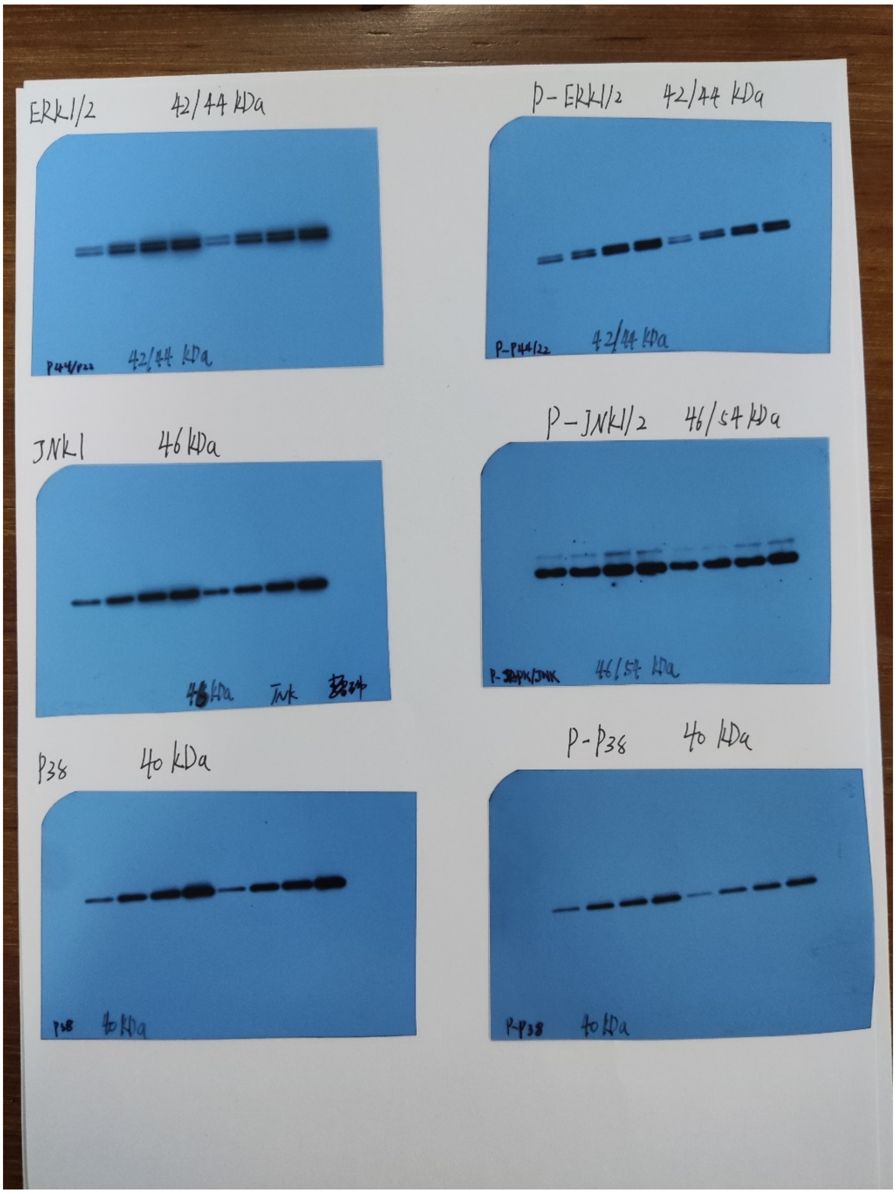

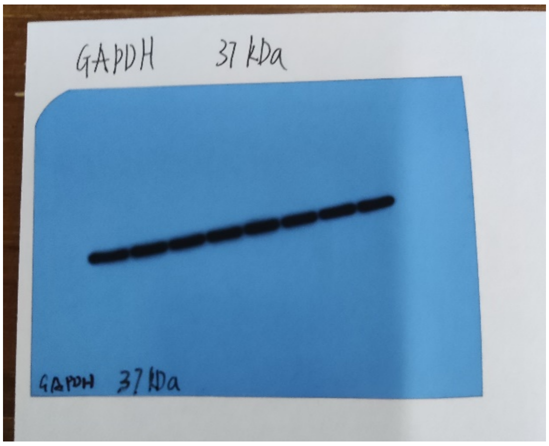

Supplement: Supplementary file 1 — Additional file 1. [file 12882_2022_2870_MOESM1_ESM.pdf]
